# Prevalence and Associated Factors of Depression among Patients with Diabetes at Jazan Province, Saudi Arabia: A Cross-Sectional Study

**DOI:** 10.1155/2019/6160927

**Published:** 2019-01-16

**Authors:** Jnadi M. Madkhali, Ammar A. Hakami, Ali H. Dallak, Ramzi M. Hakami, Abdullah A. Yatimi, Mohmmed E. Hamdi, Hisham A. Bakkari, Khalil I. Kariri, Jubran M. Abiri, Anas E. Ahmed, Abdulrahman H. Mashi

**Affiliations:** ^1^Medical Student, College of Medicine, Jazan University, Jazan, Saudi Arabia; ^2^Medical Intern, Jazan University, Jazan, Saudi Arabia; ^3^Associate Professor, Department of Community Medicine, Faculty of Medicine, Jazan University, Jazan, Saudi Arabia; ^4^Medical Resident, Internal Medicine Department, King Fahad Medical City, Riyadh, Saudi Arabia

## Abstract

**Context:**

Patients with diabetes mellitus (DM) have a poorer quality of life when compared with patients without DM. In fact, one in every five diabetic patients suffers from comorbid depression, which can lead to poor management, poor compliance with treatment, and low quality of life. Therefore, we assessed the prevalence of depression and identified its associated factors among diabetic patients at Jazan Province, KSA.

**Methods and Materials:**

A cross-sectional study was conducted among 500 diabetic patients attending a diabetic center in addition to four primary healthcare centers. We used a simple Arabic translation of the Beck Depression Inventory (BDI II) tool to evaluate the depression level among the subjects. We also evaluated the frequencies of certain sociodemographic characteristics and clinical information. Moreover, we performed univariate and multivariate analyses to identify the potential risk factors using adjusted odds ratios (AORs).

**Results:**

The prevalence of depression among DM patients was 20.6%. The majority of patients showed no depression (N = 285, 59.4%), one-fifth had mild depression (N = 96, 20.0%), some (N = 55, 11.4%) had moderate depression, and some had severe depression (N = 44, 9.2%). Depression was significantly more prevalent among uneducated patients (N = 27, 31.8%) (X^2^ = 17.627,* P *= 0.001) and patients with low monthly income (< 2500 SR/month) (N = 33, 22.8%) (X^2^ = 9.920,* P *= 0.019). Hypertension (AOR = 2.531, 95% CI [1.454, 4.406]) and ischemic heart diseases (AOR = 3.892, 95% CI [1.995, 7.593]) were considered as risk factors for depression among diabetic patients.

**Conclusions:**

Almost one in every five patients with DM is affected by depression coexisting with cardiovascular diseases. Therefore, screening for psychological problems, proper treatment, and educating patients with diabetes about DM self-management should be routine components of DM care.

## 1. Introduction

Diabetes mellitus (DM) is a growing public health concern, owing to its continuously increasing prevalence and several accompanying complications [1]. The number of adults aged 20–79 years with DM is expected to increase by 69% between 2010 and 2030 in developing countries and by 20% in developed countries [2]. The current prevalence of type 2 diabetes in the Kingdom of Saudi Arabia is 32.8%, which is estimated to reach 35.37% in 2020, 40.37% in 2025, and 45.36% in 2030 [3]. The overall prevalence of DM in Jazan Region is 12.3% [4].

DM is commonly accompanied by several serious complications such as hypoglycemia, neuropathy, retinopathy, nephropathy, and cardiovascular diseases [1]. However, the increased risk for developing depression among diabetic patients is less known [1, 5–8]. Depressed diabetic patients are less likely to participate in DM self-care activities and more likely to be physically inactive and to indulge in high-fat diet and smoking [9], which eventually lead to poor diabetes control and poor clinical outcomes [7, 10]. The prevalence of undiagnosed depression among diabetic patients is also associated with reduced quality of life and with increased healthcare costs [11]. In their RCT, Simon et al. [12] found that systematic treatment of depression in adults with DM had significantly decreased economic burden and increased depression-free time. These authors thereby concluded that depression screening and treatment should become a routine practice for DM care.

Several studies have been conducted to investigate the emotional consequences of diabetes, especially in developed countries [13]. A strong association between DM and depression has been well established in the literature [11, 13, 14]. DM was found to increase the risk of comorbid depression by two- to threefold [15, 16]. Studies from the USA and UK have reported that the prevalence of depression in patients with type 2 DM varies from 30 to 83% [17, 18]. A meta-analysis by Nouwen et al. [19] revealed that people with undiagnosed DM or impaired glucose metabolism have a significantly lower risk of depression than people with previously diagnosed type 2 DM.

Only limited data are available regarding the prevalence of depression and its associated factors among people with DM in developing countries [1]. In the Kingdom of Saudi Arabia, Al-Ghamdi [20] reported that the prevalence of depression was significantly more common among diabetic individuals (34%) than among nondiabetic individuals (13%). The author also reported that the duration of DM and poor glycemic control were significantly associated with the presence of depression. Moreover, they add that depressed diabetics suffer more than nondepressed diabetics from macrovascular complications and retinopathy. In a cross-sectional study by El Mahalli [1], the prevalence of depression among diabetic patients was found to be 49.6%, with single patients and patients with poorly controlled DM being at significantly greater risk for developing depression. Another cross-sectional study by Gemeay et al. [21] estimated depression in 37% of patients with type 1 DM and in 37.9% of patients with type 2 DM.

The aim of the present study was to assess the prevalence of depression and its associated factors among diabetic patients in a diabetic center and at four primary healthcare (PHC) centers in Jazan Province. The findings of this study are expected to bridge the gap in knowledge about depression among Saudi diabetic patients and thereby provide evidence for decision-makers to implement effective interventions, such as screening and educational programs.

## 2. Materials and Methods

### 2.1. Study Design, Settings, and Population

This is an observational, cross-sectional survey to assess the prevalence of depression and its associated factors among patients with diabetes attending a diabetic center in addition to four randomly selected primary healthcare (PHC) centers in Jazan Province during May 2017.

The inclusion criteria for this study were as follows: (1) all patients with type 1 and 2 DM who self-report being diagnosed by a healthcare worker and have a medical record at the diabetic clinic and (2) those aged 20 years and above and capable of independent communication and giving informed verbal consent. Excluded from the study were (1) patients with other types of diabetes, (2) those who were under 20 years, and (3) critically ill patients.

### 2.2. Sample Size and Sampling Procedures

First, the sample was stratified according to the two sectors, namely, rural and urban areas. Second, two PHC centers were selected randomly from each sector. A sample of 500 participants was calculated for the current study. The formula for a cross-sectional study,* n* = ([*z*^2^× p × q])/d^2^, was used to calculate the sample size, where* z* = 95% confidence interval, p = prevalence of knowledge 50%, q = 1 – p, d = error ≤5% and a 25% nonresponse rate. To adjust the number of subjects in each diabetic clinic, probability proportional to size sampling was used.

### 2.3. Data Collection and Quality Control

After obtaining informed verbal consent from participants, data were collected by the research team using a structured questionnaire translated to simple Arabic version by the study authors using back translation. Sociodemographic and clinical data were assessed. To assess comorbid depression, we used Beck Depression Inventory II (BDI) using a cut-off score of 20 or more to identify individuals with depression. The participants' responses were scored as follows: “no depression 0-13, mild depression 14-19, moderate depression 20-28, and severe depression 29-63.” [22] The current study yielded a Cronbach's alpha of 0.90 for the (BDI) items.

### 2.4. Data Management and Analysis

The data were analyzed using the Statistical Package for Social Sciences (SPSS) version 20; SPSS, Chicago, IL, USA. Data checking was done to detect any coding errors, illogical or missing values. The analysis included only complete cases without any missing values. Sociodemographic and clinical information variables, as well as the respondents' scores on the BDI II, were presented in frequencies and percentages (Tables 1, 2, and 3). To assess the association between depression and different explanatory variables, logistic regression was performed. At first, bivariate analysis was conducted for each potential risk factor; then multivariate logistic regression was done (Table 4). Adjusted odds ratios and confidence intervals were used to interpret the strength of association. The significance level was set at* p*< 0.05.

### 2.5. Ethical Consideration

The study was approved by the Department of Family Medicine, Faculty of Medicine, Jazan University, Kingdome of Saudi Arabia, and Jazan University Ethical Committee. All participants have been told that they have all rights to participate and that their information will be kept anonymous. The data collected from study participants were used only for scientific purposes.

## 3. Results

From the 500 diabetic patients surveyed, 480 agreed to take part in our study. The mean age and standard deviation of the included subjects were 49.98 ± 14.7 years (range: 20–79 years). The subjects included 307 (64%) men and 173 (36%) women. Moreover, 291 (60.6%) subjects lived in rural areas and 189 (39.4%) in urban areas. The majority of the study subjects were married (N = 347, 72.3%), followed by unmarried (N = 72, 15.0%), widowed (N = 34, 7.1%), and divorced (N = 27, 5.6%). In addition, 146 (30.4%) subjects reported a secondary education level, while 126 (26.3%) reported university level education. Some of the participants had intermediate (N = 65, 13.5%) and primary (N = 58, 12.1%) levels of education. Uneducated participants constituted 17.7% (N = 85). Overall, the median family income was 2501–5000 SR (Table 1).

Among all sociodemographic variables evaluated, only the educational level and income were found to be significantly associated with depression. Depression was significantly more prevalent among uneducated patients (N = 27, 31.8%) (X^2^ = 17.627,* P *= 0.001) and among patients with low monthly income (< 2500 SR/month) (N = 33, 22.8%) (X^2^ = 9.920,* P *= 0.019).

The survey found that the majority of subjects suffered from type 2 DM (N = 393, 81.9%), followed by those with type 1 DM (N = 87, 18.1%). Patients with DM during the past ≤ 5 years comprised the majority of the target sample (N = 183, 38.1%), followed by those with DM in the past 6–10 years (N = 159, 33.1%). Some patients had DM from 11 to 15 years (N = 80, 16.7%), while a few had DM for even > 15 years (N = 58, 12.1%). The majority of patients self-reported being compliant with DM medications (N = 398, 82.9%).

Complications of DM were self-reported by participants and presented in Table 2; 47% (N = 228) suffered from comorbid retinopathy, 41.3% (N = 198) from neuropathy, 41.3% (N = 198) from hypertension, 15.0% (N = 72) from nephropathy, and 11.2% (N = 54) from ischemic heart diseases.

The prevalence of self-reported clinical depression (Beck Depression Inventory (BDI) score ≥20) among patients with diabetes was 20.6%. The majority of patients did not have depression (N = 285, 59.4%), one-fifth had mild depression (N = 96, 20.0%), some (N= 55, 11.4%) had moderate depression, and some (N = 44, 9.2%) had severe depression (Table 3).

Multivariate regression revealed hypertension (adjusted odds ratio (AOR) = 2.531, 95% CI [1.454, 4.406]) and ischemic heart disease (AOR = 3.892, 95% CI [1.995, 7.593]) as risk factors for depression. These results indicate that diabetics with a comorbid ischemic heart disease and hypertension were 3.8 and 2.5 times more likely to suffer from depression, respectively. In addition, patients with type 1 DM were 2.249 times more likely to suffer from depression (AOR = 2.249, 95% CI [1.043, 4.849]). Other factors such as having nephropathy (crude odds ratio (COR) = 2.096, 95% CI [1.183, 3.712]) and neuropathy (COR = 1.653, 95% CI [1.027, 2.602]) revealed a crude positive association with depression but did not persist after controlling for sociodemographic factors (Table 4).

## 4. Discussion

The present study was to assess the prevalence of depression and its associated factors in diabetic patients attending PHC centers. Authors revealed that 20.6% of patients with types 1 and 2 DM suffer from depression. These results are consistent with those of prior studies from United States (17.5%) and Ethiopia (17%) [23, 24]. However, our values are lower than those reported by prior studies from the Kingdom of Saudi Arabia, Bahrain, Palestine, and Pakistan [1, 8, 20, 25, 26]. The possible explanations for the difference in the results may include differences in the sample size, screening tools used, different cut-off scores employed, and sociodemographic status.

Depression was found to be related to illiteracy and low levels of income in this study. Some past studies have also reported similar findings [1, 23, 27–29]. This education-income-depression relationship can be explained by relating the education level to the economic condition of people, as income and job may account for the presence of depression among poorly educated individuals [30]. Moreover, highly educated people are less likely to be depressed because they can attain better jobs and have greater healthcare resources than less educated people [30].

Hypertension and ischemic heart diseases were identified as risk factors for depression in this study. This finding is supported by that of previous studies that linked other physical illnesses such as cardiovascular diseases to depression [20, 25, 27, 31–33]. For instance, Burgic-Radmanovic et al. [33] found comorbidity of depression and hypertension in 38.5% of patients and thereby concluded that cardiovascular diseases often coexist with depression, which in turn is associated with cognitive impairment and higher functional disability that can lead to further suffering, worse health conditions, difficult treatment, and poor outcomes [33, 34].

## 5. Limitations

The present study has some limitations. First, it is a cross-sectional study and cannot establish the causal relationship between DM and depression. Second, the data were gathered from participants through their self-report of depressive symptoms, DM complications, and compliance with medication; thus, a clinical interview may be better because of its higher level of specificity. Third, as the study was conducted in one province of KSA (Jazan Province), its results are representative only for the province population not for the whole country.

## 6. Conclusion

Depression in this study is highly prevalent. Almost one in every five patients with diabetes is affected by depression coexisting with cardiovascular diseases. Moreover, depression is more prevalent among uneducated patients and patients with low levels of income. Therefore, screening for psychological problems, proper treatment, and educating patients with diabetes about DM self-management should be routine components of DM care.

## Figures and Tables

**Table 1:** Sociodemographic characteristics of diabetic patients, Jazan, May 2017 (N = 480).

**Variable**	**Category**	**N**	%
Gender	Male	307	64.0
	Female	173	36.0
Age groups (years)	20-29	52	10.8
	30-39	60	12.5
	40-49	107	22.3
	50-59	116	24.2
	60-79	145	30.2
Residency	Urban	189	39.4
	Rural	291	60.6
Marital status	Single	72	15.0
	Married	347	72.3
	Divorced	27	5.6
	Widowed	34	7.1
Level of education	University	126	26.3
	Secondary	146	30.4
	Intermediate	65	13.5
	Primary	58	12.1
	Uneducated	85	17.7
Income	> 10000 SR	106	22.1
	5001-10000 SR	113	23.5
	2501-5000 SR	116	24.2
	0-2500 SR	145	30.2

**Table 2:** Disease information of diabetic patients, Jazan, May 2017 (N = 480).

Variable	Category	N	%
Type of DM	Type 1	87	18.1
	Type 2	393	81.9
Duration of DM	0-5 Years	183	38.1
	6-10 Years	159	33.1
	11-15 Years	80	16.7
	> 15 years	58	12.1
Compliance to medications	Yes	398	82.9
	No	82	17.1
Comorbidities	Retinopathy	228	47.5
	Nephropathy	72	15.0
	Neuropathy	198	41.3
	Hypertension	198	41.3
	Ischemic Heart Disease	54	11.2

**Table 3:** Summary of BDI II scores and their interpretation.

BDI II scores	Interpretation	N	%
0-13	Non depressed	285	59.4
14-19	Mild depression	96	20.0
20-28	Moderate depression	55	11.4
29-63	Severe depression	44	9.2

BDI: Beck Depression Inventory

**Table 4:** Multivariate analysis of different factors and depression among diabetic patients, Jazan, May 2017 (N = 480).

Factors	Depression		COR (95% CI)	AOR (95% CI)	*P *value
	Yes	No			
Type of DM					
Type 1 DM	19	68	1.312 (0.741, 2.323)	2.249 (1.043, 4.849)^*∗*^	0.039
Type 2 DM	69	324			
Duration of DM					
0-5 Years	28	155	0.401 (0.202, 0.798)	0.752 (0.312, 1.814)	0.526
6-10 Years	29	130	0.496 (0.249, 0.985)	0.828 (0.367, 1.868)	0.649
11-15 Years	13	67	0.431 (0.191, 0.973)	0.585 (0.241, 1.419)	0.236
> 15 years	18	40			
Compliance to DM medications					
Yes	70	328	0.759 (0.424, 1.359)	0.958 (0.495, 1.857)	0.900
No	18	64			
Retinopathy					
Yes	43	185	1.069 (0.673, 1.698)	0.607 (0.331, 1.115)	0.108
No	45	207			
Nephropathy					
Yes	21	51	2.096 (1.183, 3.712)	1.384 (0.707, 2.709)	0.343
No	67	341			
Neuropathy					
Yes	45	153	1.635 (1.027, 2.602)	1.149 (0.612, 2.158)	0.666
No	43	239			
Hypertension					
Yes	53	145	2.580 (1.606, 4.142)	2.531 (1.454, 4.406)^*∗*^	0.001
No	35	247			
Ischemic heart disease					
Yes	24	30	4.525 (2.486, 8.236)	3.892 (1.995, 7.593)^*∗*^	0.000
No	64	362			

COR: crude odds ratio, AOR: adjusted odds ratio, CI: confidence interval,*∗*  *P*< 0.05

## Data Availability

The data used to support the findings of this study are available from the corresponding author upon request.
